# The efficacy and safety of concentrated herbal extract granules, YH1, as an add-on medication in poorly controlled type 2 diabetes: A randomized, double-blind, placebo-controlled pilot trial

**DOI:** 10.1371/journal.pone.0221199

**Published:** 2019-08-15

**Authors:** Yueh-Hsiang Huang, Szu-Tah Chen, Feng-Hsuan Liu, Sheng-Hwu Hsieh, Chia-Hung Lin, Miaw-Jene Liou, Chih-Ching Wang, Chung-Huei Huang, Geng-Hao Liu, Jr-Rung Lin, Lan-Yan Yang, Tzu-Yang Hsu, Ming-Chung Lee, Chun-Teng Huang, Yi-Hong Wu

**Affiliations:** 1 Division of Chinese Internal Medicine, Center for Traditional Chinese Medicine, Chang Gung Memorial Hospital, Taoyuan, Taiwan; 2 Graduate Institute of Clinical Medical Sciences, College of Medicine, Chang Gung University, Taoyuan, Taiwan; 3 Division of Endocrinology and Metabolism, Department of Internal Medicine, Chang Gung Memorial Hospital, Linkou, Taiwan; 4 School of Traditional Chinese Medicine, College of Medicine, Chang Gung University, Taoyuan, Taiwan; 5 Clinical Informatics and Medical Statistics Research Center and Graduate Institute of Clinical Medicine, Chang Gung University, Taoyuan, Taiwan; 6 Biostatistics and Informatics Unit, Clinical Trial Center, Chang Gung Memorial Hospital and Chang Gung University, Taoyuan, Taiwan; 7 Brion Research Institute of Taiwan, Taipei City, Taiwan; 8 Sanford Burnham Prebys Medical Discovery Institute, La Jolla, California, United States of America; Tabriz University of Medical Sciences, ISLAMIC REPUBLIC OF IRAN

## Abstract

**Background:**

In Asian countries, many patients with type 2 diabetes fail to achieve controlled glycated hemoglobin (HbA_1c_) levels while taking several classes of oral hypoglycemic agents (OHAs). Traditional Chinese medicine could be an alternative therapeutic option for poorly controlled type 2 diabetes. YH1 is a concentrated Chinese herbal extract formula that combines *Rhizoma Coptidis* and Shen-Ling-Bai-Zhu-San. This randomized, double-blind, placebo-controlled pilot study evaluated YH1 as an add-on medication for poorly controlled type 2 diabetes.

**Methods:**

Forty-six patients with poorly controlled type 2 diabetes were randomly assigned 1:1 to the YH1 or placebo group. Before the trial, all subjects had received three or more classes of OHAs with HbA_1c_ > 7.0% (53 mmol/mol) and a body mass index ≥ 23 kg/m^2^. During the 12-week trial, participants continued to take OHAs without any dose or medication changes. The primary endpoint was the percentage change in HbA_1c_ level. Per-protocol analysis was applied to the final evaluation.

**Results:**

At week 12, there was an 11.1% reduction in HbA_1c_ from baseline and a 68.9% increase in homeostatic model assessment (HOMA) of β cell function in the YH1 group, which also exhibited significant reductions in two-hour postprandial glucose (-26.2%), triglycerides (-29.5%), total cholesterol (-21.6%), low-density lipoprotein cholesterol (-17.4%), body weight (-0.5%), and waist circumference (-1.1%). The changes in fasting plasma glucose, HOMA insulin resistance and symptom scores were not significantly different between the YH1 and placebo groups. No serious adverse events occurred during this clinical trial.

**Conclusions:**

This pilot study indicates that YH1 together with OHAs can improve hypoglycemic action and β-cell function in overweight/obese patients with poorly controlled type 2 diabetes. YH1 is a safe add-on medication for OHAs and has beneficial effects on weight control and lipid metabolism. A larger study population with longer treatment and follow-up periods is required for further verification.

## Introduction

Diabetes, especially type 2 diabetes mellitus, is a serious, chronic metabolic disease that affects patients worldwide, and its prevalence has steadily increased in recent decades [1]. According to the International Diabetes Federation (IDF) guidelines [2], achieving an HbA_1c_ below 7.0% (53 mmol/mol) minimizes the risk of developing diabetes-related complications. Previous studies have shown that fewer than one-third of patients in China with type 2 diabetes taking oral antidiabetic medications achieved the goal of HbA_1c_ < 7.0% (53 mmol/mol) [3]. In addition, the majority of patients with type 2 diabetes in Taiwan also exhibited unsatisfactory glycemic control, which was associated with diabetes complications [4]. Currently, numerous patients in Taiwan who take three or more oral hypoglycemic agents (OHAs) cannot effectively control their HbA_1c_ levels. The failure of OHA treatment in patients with type 2 diabetes is defined as treatment-resistant type 2 diabetes, although the notion of “resistant diabetes” is not commonly used [5]. Insulin injection is the next available option to treat resistant diabetes; however, hypoglycemia and weight gain are common discouraging side effects. Thus, there is an urgent need to develop new drugs to reverse poorly controlled type 2 diabetes.

Traditional Chinese medicine (TCM) has played a role in the treatment of type 2 diabetes since ancient times [6] and is an excellent resource for discovering innovative medicines. The therapeutic effects of herbal medicines for type 2 diabetes have been published in China [7, 8]. However, a population-based study in Taiwan reported that while 77.9% of patients with type 2 diabetes utilized TCM, only 13.9% of them used it to treat diabetes [9]. *Rhizoma Coptidis* was first recorded during the eastern Han dynasty (25–220 AD), and it has been prescribed by Chinese herbalists to treat diabetes-related symptoms for more than 2000 years. Modern pharmacological research has identified the major chemical constituents of *Rhizoma Coptidis* as alkaloids, including berberine, coptisine, worenine, palmatine, jatrorrhizine, and epiberberine [10]. Among these, berberine is generally considered the primary contributor to the main bioactivities of this herb, including its antidiabetic, antibiotic, antioxidant, anti-inflammatory, antidyslipidemia and antiobesity activities [11]. Berberine is considered an AMP-activated protein kinase (AMPK) activator, an inducer of glucose transporter-4 and insulin receptor mRNA expression, an α-glucosidase inhibitor, a glucagon-like peptide-1 (GLP-1) inducer, and an inhibitor of mitochondrial respiratory chain complex I, resulting in the stimulation of glycolysis [11–13]. *Radix Ginseng*, *Rhizoma Dioscoreae*, *Rhizoma Atractylodis macrocephalae*, and *Poria* are four major herbs of Shen-Ling-Bai-Zhu-San (SLBZS). These herbs exert antidiabetic effects *in vitro* and *in vivo* by enhancing insulin production/secretion, modulating antioxidant activities and inflammatory pathways, promoting the release of GLP-1, increasing glucose metabolism/uptake, or improving energy metabolism in skeletal muscle [14–17]. Therefore, *Rhizoma Coptidis* and SLBZS together should provide additive effects in treating poorly controlled type 2 diabetes.

Although decoction is a common methodology for preparing herbal medicines, it is inconvenient and difficult to perform with consistent quality. Using concentrated herbal extract granules to treat resistant diabetes appears more applicable, but the safety and efficacy require validation in controlled clinical trials. In addition, Chinese herbalists may choose *Rhizoma Coptidis* or SLBZS to treat diabetes-related symptoms, but the combination of both medicines for diabetes treatment has not been studied previously. To create an innovative antidiabetic medication and avoid decoction procedures, YH1 was designed as concentrated herbal granules containing *Rhizoma Coptidis* and SLBZS. The aim of this pilot trial was to evaluate the safety and efficacy of YH1 as an add-on medication for poorly controlled type 2 diabetes.

## Materials and methods

### Trial design

This study was a randomized, double-blind, placebo-controlled trial. Patients who met the following inclusion criteria were referred from endocrinology and metabolism clinics to Chinese medicine clinics to join this study: 1) type 2 diabetes; 2) treatment with ≥ 3 classes of OHAs with persistent (> 6 months) poorly controlled glycemia (HbA_1c_ >7.0% or 53 mmol/mol); 3) aged 20–75 years; and 4) body mass index (BMI) ≥ 23 kg/m^2^.

The exclusion criteria were as follows: 1) type 1, gestational, or other specific types of diabetes; 2) insulin therapy within the past three months; 3) serious gastrointestinal (GI) tract diseases, including peptic ulcers and GI tract bleeding; 4) history of stressful situations, including diabetic ketoacidosis, nonketotic hyperosmolar diabetic coma, severe infection, or surgery in the previous month; 5) hepatic insufficiency with alanine aminotransferase (ALT) >72 U/L or renal insufficiency with an estimated glomerular filtration rate (eGFR) < 60 mL/min/1.73 m^2^; 6) uncontrolled hypertension (blood pressure ≥ 160/100 mmHg); 7) mental illness; 8) abuse of or addiction to alcohol, psychoactive substances or other drugs; 9) pregnancy, lactation, or plan to become pregnant; 10) hemoglobin disease or chronic anemia; 11) underlying conditions that could lead to poor compliance; 12) history of cerebrovascular disease or myocardial infarction; and 13) Chinese medicine treatment within the past two weeks.

**Fig 1:**
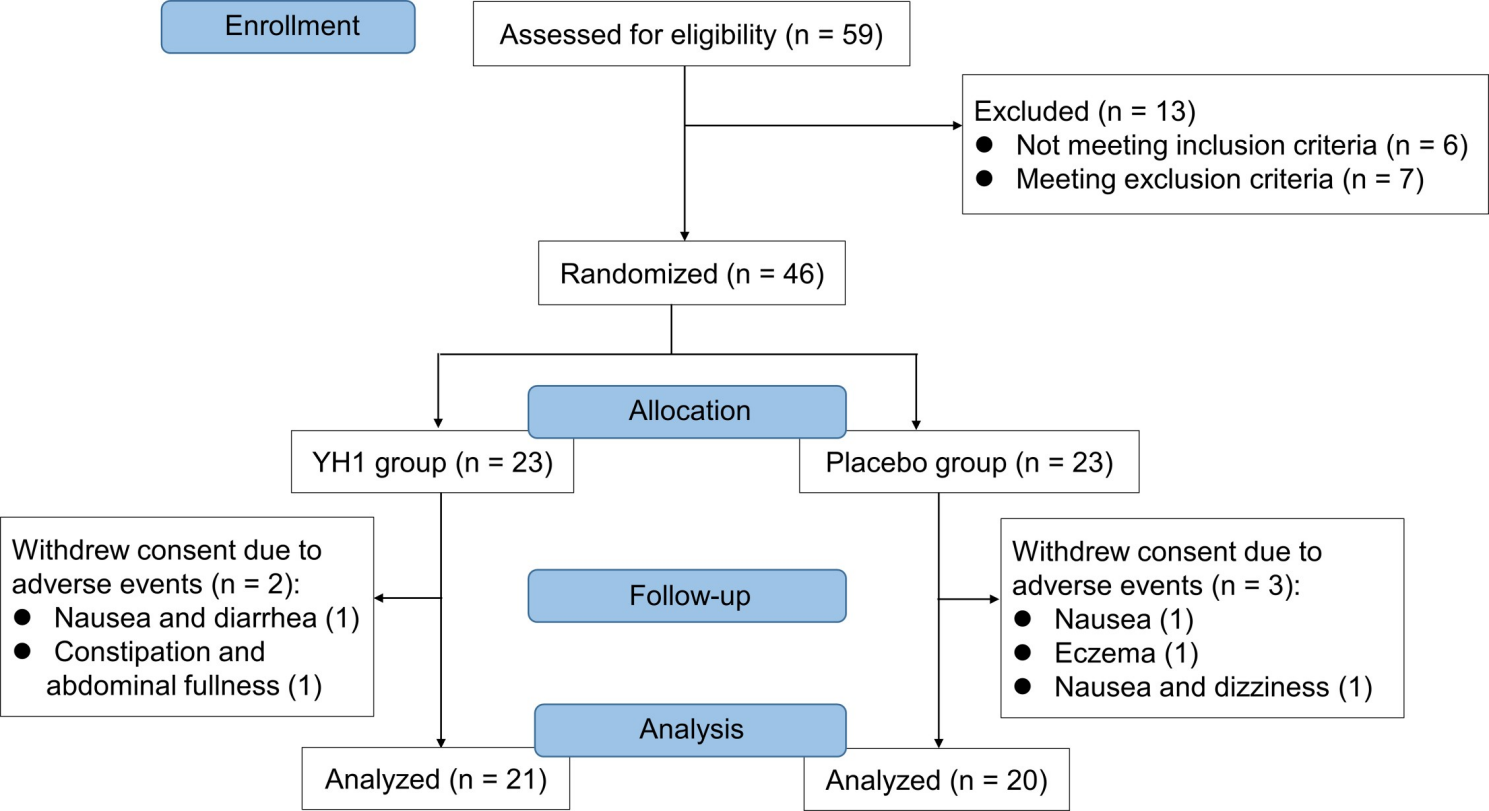
CONSORT flow diagram of enrollment, randomization, and treatment. Two patients in the YH1 group and three patients in the placebo group were excluded from the study for the various reasons listed.

The trial protocol was approved by the Committee on Research Ethics of Chang Gung Memorial Hospital in Taiwan in January 2016 (No. 104-7934A3) with a project duration from March 2016 to February 2018. This clinical study was registered at ClinicalTrials.gov (NCT02752880) in April 2016. After clinical trial registration, the first participant was enrolled into this study in June 2016. Written informed consent was obtained from all eligible subjects before participation. Participants were randomly assigned 1:1 to receive either YH1 or placebo for 12 consecutive weeks (Fig 1). Subjects in the two groups orally ingested two packages of granules (3 g/package) three times daily with warm water after a meal. The dose of the OHAs was stable for at least three months before enrollment and remained unchanged throughout the study. All subjects were instructed to maintain their lifestyle habits during the 12-week study period. Because of difficulties in enrolling participants, the eligibility criteria were expanded once: the inclusion criteria for HbA_1c_ and BMI were reduced from ≥ 9% to > 7% and from ≥ 24 kg/m^2^ to ≥ 23 kg/m^2^, respectively. Furthermore, one additional branch of the hospital was added for subject recruitment. The Committee on Research Ethics of Chang Gung Memorial Hospital in Taiwan also approved the protocol amendment (Nos. 105-7009C, 106-0917C). The study was completed in February 2018. The CONSORT checklist and trial protocol are available as supporting information (S1 CONSORT checklist, S1 Protocol). The authors confirm that all ongoing and related trials for this drug/intervention are registered.

During the 12-week trial period, patients were assessed at 0, 2, 4, 8 and 12 weeks. Baseline characteristic data, including age, sex, duration of diabetes, smoking or alcohol consumption, other chronic diseases, and medications, were acquired at the first visit. In each session, all subjects underwent a symptom assessment [7] (S1A Table) and a physical examination including body weight, BMI and waist circumference. In addition, drug compliance and adverse events were recorded by project staff. HbA_1c_, fasting plasma glucose (FPG), and 2-hour postprandial glucose (2hPG) were measured at 0, 4 and 12 weeks. The insulin resistance index, β-cell function index, lipid profile, and hepatic and renal function were assessed at 0 and 12 weeks.

### Study medication

**Fig 2:**
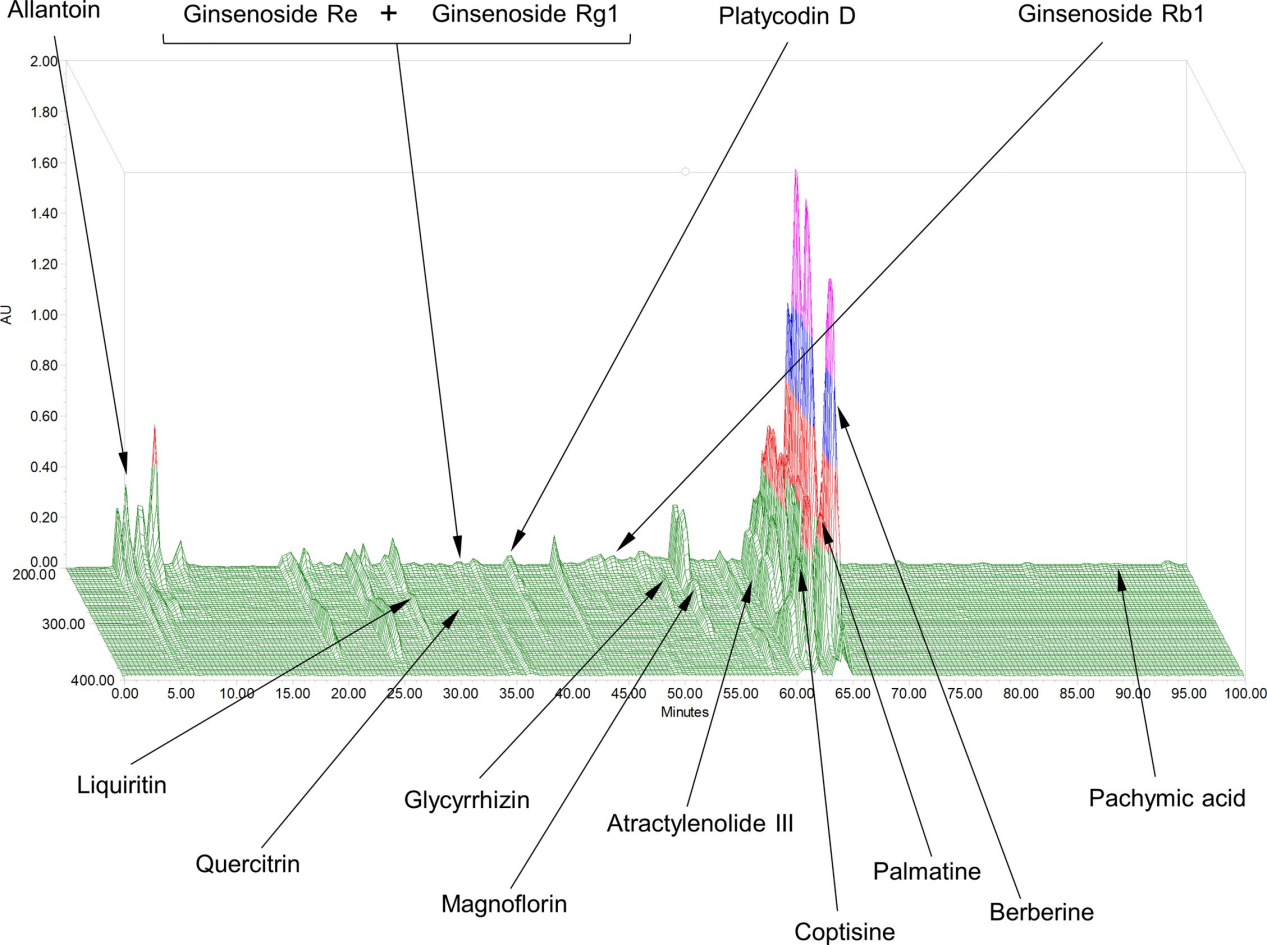
3D-HPLC fingerprint of YH1. The chemical composition of YH1 was analyzed using high-performance liquid chromatography (HPLC) with a photodiode array (PDA). Fourteen components, including allantoin, atractylenolide III, berberine, coptisine, ginsenoside Rb1, ginsenoside Re, ginsenoside Rg1, glycyrrhizin, liquiritin, pachymic acid, palmatine, platycodin D, magnoflorin and quercitrin, were detected.

YH1 with one batch number was manufactured by Sun Ten Pharmaceutical Co., Ltd., a renowned manufacturer of concentrated herbal extract granules conforming to the standards of good manufacturing practices (GMP) in New Taipei City, Taiwan. Each concentrated herbal extract granule of YH1 contains 50% *Rhizoma Coptidis* and 50% SLBZS. *Rhizoma Coptidis* and SLBZS have already been approved by the Ministry of Health and Welfare in Taiwan as ethical drugs. The botanical origins of the study medications are listed in Table 1. YH1 granules were packed in aluminum foil packages. The placebo was also prepared as granules by Sun Ten Pharmaceutical Co., Ltd., and the packaging of the placebo was identical to that of YH1. The chemical composition of YH1 was analyzed and profiled by using high-performance liquid chromatography (HPLC) with photodiode array (PDA) detection (S1 Fig). Fourteen components, namely, allantoin, atractylenolide III, berberine, coptisine, ginsenoside Rb1, ginsenoside Re, ginsenoside Rg1, glycyrrhizin, liquiritin, pachymic acid, palmatine, platycodin D, magnoflorin and quercitrin, were simultaneously used in the qualitative analysis by the developed HPLC-PDA method. In addition, a 3D-HPLC fingerprint analysis of YH1 was obtained (Fig 2). The quantitative measurement indicated that one gram of YH1 contained 20.05 mg of berberine.

**Table 1:** The composition and botanical origin of YH1.

YH1	Crude drugs	English name	Botanical origin
***Rhizoma Coptidis* (50%)**		Huang Lian	*Coptis chinensis* Franch, rhizome
**Shen-Ling-Bai-Zhu-San (50%)**	*Radix Ginseng*	Ren Shen	*Panax ginseng* C.A.Mey., root
	*Poria*	Fu Ling	*Poria cocos* (Schw.) Wolf, inner parts of the sclerotia
	*Rhizoma Atractylodis macrocephalae*	Bai Zhu	*Atractylodes* *macrocephala* Koidz., rhizome
	*Semen Lablab album*	Bai Bian Dou	*Dolichos lablab* L., seed
	*Rhizoma Dioscoreae*	Shan Yao	*Dioscorea opposita* Thunb., rhizome
	*Embryo Nelumbinis*	Lian Zi Rou	*Nelumbo nucifera* Gaertn., seed
	*Radix Platycodonis*	Jie Geng	*Platycodon grandiflorus* (Jacq.) A.DC., root
	*Semen Coicis*	Yi Yi Ren	*Coix lacryma-jobi* L. var. *ma-yuen* (Roman.) Stapf, seed
	*Fructus Amomi *	Sha Ren	*Amomum* *villosum* Lour., fructus
	*Fructus Jujubae*	Da Zao	*Ziziphus* *jujuba* Mill., fructus
	*Radix Glycyrrhizae*	Gan Cao	*Glycyrrhiza uralensis* Fisch., root and rhizome

YH1 contains *Rhizoma Coptidis* (50%) and Shen-Ling-Bai-Zhu-San (SLBZS) (50%). SLBZS consists of Radix Ginseng, Poria, Rhizoma Atractylodis macrocephalae, Semen Lablab album, Rhizoma Dioscoreae, Embryo Nelumbinis, Radix Platycodonis, Semen Coicis, Fructus Amomi, Fructus Jujubae, and Radix Glycyrrhizae at a 3:3:3:2.3:3:1.5:1.5:1.5:1.5:1.5:3 ratio. The abovementioned materials were manufactured by Sun Ten Pharmaceutical Company, which is a licensed GMP pharmaceutical factory in New Taipei City, Taiwan. Quality control factors included harvest location, macroscopic and microscopic identification, loss on drying, total ash, acid-insoluble ash, water soluble extract, dilute ethanol soluble extract, thin layer chromatography, marker substance assay, residual pesticides, heavy metals, and microbiological contaminants. The standard YH1 manufacturing procedure is a continuous process involving the aqueous extraction of medicinal raw materials with boiled water, filtration, concentration, and drying (fluid-bed granulation).

### Randomization and allocation concealment

Randomization codes were generated by an independent statistician using SAS software (version 9.2, Cary, NC, USA). Study drugs prepared by Sun Ten Pharmaceutical Co., Ltd. were packed and numbered according to the random coding form. The random coding form was concealed in an opaque envelope after randomization. The envelope was not decoded until the end of the trial. Study drugs were provided based on the assigned numbers according to the visit sequence and study drug number sequence. During the trial, neither the clinicians nor the patients were aware of the grouping.

### Outcome assessment

The primary efficacy endpoint was the percentage change in HbA_1c_ levels from baseline to 12 weeks. The HbA_1c_ level was measured by HPLC in the Department of Laboratory Medicine at Chang Gung Memorial Hospital. The secondary efficacy endpoints were the percentage changes in parameters including FPG and 2hPG levels, homeostatic model assessment (HOMA) of insulin resistance (IR), HOMA-β, lipid profile, body weight and waist circumference and in clinical symptoms from baseline to 12 weeks.

Regarding safety assessment, vital signs were recorded at every visit. Laboratory tests including ALT and serum creatinine were performed before and after the trial. Participants were encouraged to report any discomfort to the project manager throughout the trial. Adverse events including any undesirable or unintended symptoms were assessed regardless of the causal relationship with the study drug. All adverse events were recorded on the case report form with footnotes describing the time of onset, resolution and severity of symptoms. The severity of adverse events was reported by grading on a scale of 1 to 5 according to the Common Terminology Criteria for Adverse Events (CTCAE, Version 4.0).

### Sample size estimation

Based on previous clinical practice using YH1 to treat patients with poorly controlled type 2 diabetes (unpublished data), the percentage change in HbA_1c_ from baseline was a 14.3% decrease after 3 months of treatment. Therefore, a two-proportion test given by a change rate of 0.1% for placebo and 14.3% for YH1 with a significance level of 0.05 was applied. A statistical power of 0.7 was desirable because this study was a pilot trial, and it was assumed that only a small group of patients with poorly controlled type 2 diabetes would meet the study criteria. The sample size estimation before this study was twenty-five subjects per group using G_Power (version 3.1). For a 30% expected dropout rate, a total of 80 enrollments in a 1:1 ratio between the YH1 and placebo groups were planned in the clinical trial protocol.

### Statistical analysis

The efficacy analysis was based on the per-protocol analysis set of 41 subjects (21 in the YH1 group and 20 in the placebo group). The safety analysis was based on records from 46 subjects receiving treatment. The comparison of clinical characteristics between the two groups was based on the Mann–Whitney U test for continuous variables. Fisher’s exact test was used to evaluate the associations between categorical variables. To analyze changes in parameters compared with their baseline values, we generated boxplots as a nonparametric method of analysis and used the Wilcoxon signed-rank test for paired difference comparisons. All of the statistical analyses were performed in R V.3.4.3 (http://www.r-project.org). A two-tailed *p* <0.05 was considered significant.

## Results

### Subject characteristics

From March 2016 to February 2018, 59 patients participated in the initial screening, and 46 eligible subjects entered the trial. After treatment, 5 subjects (2 in the YH1 group and 3 in the placebo group) withdrew consent during the trial because of adverse events and were thus excluded from the study (Fig 1). This study was completed with 46 participants because the YH1 study medication, as a concentrated Chinese herbal extract, had a two-year shelf life. It was difficult to enroll additional subjects because this trial was strictly for patients with poorly controlled type 2 diabetes, and these patients could receive insulin injection as a standard treatment. In addition, YH1 could be prescribed in clinical practice and reimbursed by national health insurance in Taiwan, which further reduced the motivation of patients to participate in clinical trials. In the end, 41 subjects, including 21 in the YH1 group and 20 in the placebo group, completed the 12-week study. There were 30 patients receiving three classes of OHAs, 10 patients receiving four OHAs, and 1 patient receiving five OHAs (S2 Table). Three participants smoked without addiction, and six participants were casual drinkers; there were no significant differences in these variables between the YH1 and placebo groups. Subjects did not change these habits during the 12-week study period. Based on the Mann–Whitney U test or Fisher’s exact test, no significant differences were observed between the two groups at baseline with respect to anthropometric, biochemical, and clinical parameters (Table 2).

**Table 2:** Baseline characteristics of participants (per-protocol analysis).

		YH1 Group (*n* = 21)	Placebo Group (*n* = 20)	*p* value
		median (min, max)	median (min, max)	
Demographic characteristics				
Age (yr)		50.0 (33.0, 69.0)	56.0 (40.0, 66.0)	.44
Sex, male / female		13 / 8	8 / 12	.22
Diabetes duration (yr)		10.0 (1.0, 23.0)	11.5 (4.0, 20.0)	.24
Number of OHAs				.60
	Three	14	16	
	Four	6	4	
	Five	1	0	
Anthropometric characteristics				
Weight (kg)		73.5 (59.6, 107.0)	72.2 (60.9, 88.1)	.67
Body mass index (kg/m^2^)		26.1 (23.4, 35.4)	28.0 (23.0, 36.0)	.48
Waist circumference (cm)		90.5 (80.5, 110.5)	93.0 (81.0, 105.0)	.19
Blood pressure and heart rate				
Systolic BP (mmHg)		131.0 (107.0, 157.0)	123.5 (110.0, 153.0)	.16
Diastolic BP (mmHg)		76.0 (64.0, 92.0)	73.5 (65.0, 93.0)	.73
Heart rate (beat/min)		82.0 (63.0, 104.0)	82.0 (65.0, 117.0)	.78
Antihypertensive drug, no. (%)		10 (47.6)	8 (40.0)	.76
Laboratory data				
HbA_1c_ (%)		8.1 (7.5, 11.3)	8.7 (7.6, 11.8)	.19
FPG (mg/dL)		159.0 (85.0, 243.0)	159.0 (105.0, 283.0)	.77
2hPG (mg/dL)		220.2 (117.0, 371.0)	216.5 (120.0, 357.0)	.95
Fasting insulin (μU/mL)		8.0 (2.9, 15.7)	6.9 (2.9, 11.9)	.25
HOMA-IR		3.3 (0.8, 6.1)	3.1 (0.8, 5.5)	.51
HOMA-β		31.7 (8.0, 152.8)	25.6 (10.3, 48.7)	.27
ALT (U/L)		24.0 (8.0, 68.0)	25.0 (8.0, 53.0)	.38
Creatinine (mg/dL)		0.7 (0.4, 1.1)	0.6 (0.4, 1.0)	.55
Total cholesterol (mg/dL)		176.0 (139.0, 232.0)	187.0 (114.0, 254.0)	.41
HDL-C (mg/dL)		43.0 (31.0, 66.0)	42.5 (28.0, 61.0)	.44
LDL-C (mg/dL)		103.0 (58.0, 164.0)	117.0 (60.0, 192.0)	.25
Triglyceride (mg/dL)		151.0 (75.0, 446.0)	161.0 (53.0, 596.0)	.99
Hypolipidemic drug, no. (%)		10 (47.6)	8 (40.0)	.76
Score of TCM symptoms of diabetes		5.0 (1.0, 14.0)	6.0 (2.0, 13.0)	.43

Statistics were evaluated by using the Mann–Whitney U test for continuous variables and Fisher’s exact test for categorical variables. No significant differences were observed between the two groups at baseline. Because of the small sample sizes, the data are presented as the median (min, max). OHAs, oral hypoglycemic agents; BP, blood pressure; HbA_1c_, glycated hemoglobin; FPG, fasting plasma glucose; 2hPG, 2-hour postprandial glucose; HOMA-IR, homeostatic model assessment of insulin resistance; HOMA-β, homeostatic model assessment of β cell function; ALT, alanine aminotransferase; HDL-C, high-density lipoprotein cholesterol; LDL-C, low-density lipoprotein cholesterol; TCM, traditional Chinese medicine.

### Primary and secondary outcomes

**Fig 3:**
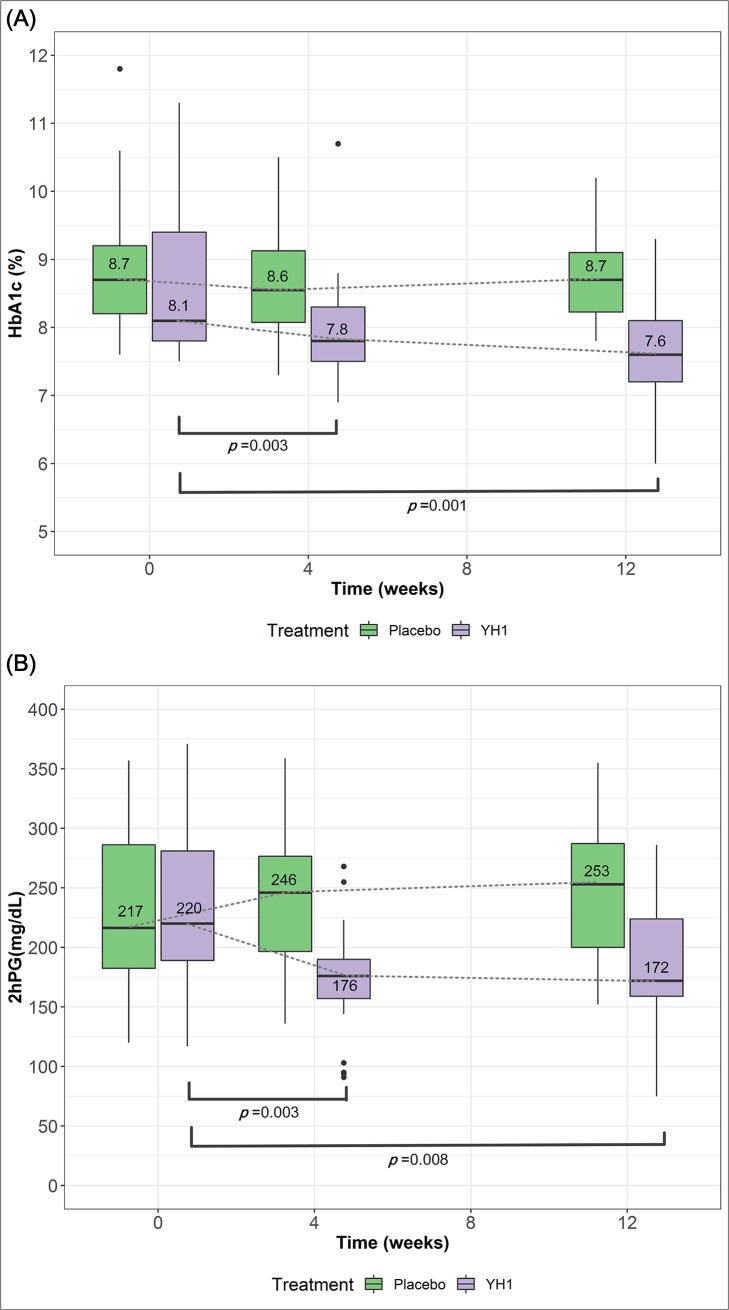
Primary outcomes (HbA_1c_) and secondary outcomes (2hPG) over 12 weeks. The distribution of data (median, quartiles, range and outliers) for HbA_1c_ (A) and 2hPG (B) in the YH1 and placebo groups at week 0, week 4, and week 12 are displayed in the boxplot. To analyze the changes in parameters from their baseline values, Wilcoxon signed-rank test was applied. A value of *p* < 0.05 was considered significant. In the boxplot (Fig 3A), there was a significant reduction in the median HbA_1c_ value at week 4 (*p* = 0.003) and week 12 (*p* = 0.001) compared with the value at baseline in the YH1 group. In addition, the median 2hPG level from week 0 to week 12 declined significantly from 220 mg/dL to 172 mg/dL in the YH1 group (Fig 3B, *p* = 0.008). HbA_1c_, glycated hemoglobin; 2hPG, 2-hour postprandial glucose.

Primary and secondary outcomes are summarized in Table 3. At week 12, the median HbA_1c_ in the YH1 group was 7.6% (60 mmol/mol), with a range of 6.0–9.3% (42–78 mmol/mol). The median HbA_1c_ in the placebo group was 8.7% (72 mmol/mol), with a range of 7.8–10.2% (62–88 mmol/mol). From week 0 to 12, the HbA_1c_ level decreased by 11.1% in the YH1 group, while no change was observed in the placebo group (*p* = 0.008). The distribution of series values of HbA1c (A) and 2hPG (B) in the YH1 and placebo groups at week 0, week 4, and week 12 are shown in the boxplots (Fig 3). The trends of reduction in HbA_1c_ and 2hPG levels were consistent throughout YH1 treatment.

**Table 3:** Outcomes and percentage changes in parameters from baseline to 12 weeks.

	YH1 Group	Placebo Group	*p* value
	(*n* = 21)	(*n* = 20)	
	median (min, max)	median (min, max)	
**Weight (kg)**			
At week 12	73.6 (57.7, 108.2)	74.3 (60.9, 87.6)	
Relative change (%)	-0.5 (-3.2, 1.7)	0.6 (-1.7, 4.2)	0.030*
**Waist circumference (cm)**			
At week 12	90.5 (79.0, 112.5)	93.3 (81.5, 105.5)	
Relative change (%)	-1.1 (-4.1, 2.2)	0.5 (-2.8, 3.2)	0.012*
**Body mass index (kg/m**^**2**^**)**			
At week 12	26.1 (23.1, 35.6)	28.3 (22.8, 35.5)	
Relative change (%)	-0.4 (-3.3, 1.7)	0.4 (-1.8, 4.0)	0.083
**HbA**_**1c**_ **(%)**			
At week 12	7.6 (6.0, 9.3)	8.7 (7.8, 10.2)	
Relative change (%)	-11.1 (-30.2, 14.1)	0.0 (-20.4, 7.3)	0.008**
**FPG (mg/dL)**			
At week 12	136.0 (83.0, 223.0)	167.5 (112.0, 261.0)	
Relative change (%)	-12.0 (-54.1, 51.7)	5.0 (-27.3, 72.4)	0.066
**2hPG (mg/dL)**			
At week 12	172.0 (75.0, 286.0)	253.0 (152.0, 355.0)	
Relative change (%)	-26.2 (-60.3, 94.1)	5.1 (-43.0, 90.6)	0.006**
**Fasting insulin (μU/mL)**			
At week 12	8.8 (2.7, 21.7)	6.1 (2.8, 14.5)	
Relative change (%)	12.9 (-46.1, 185.5)	1.0 (-55.1, 69.4)	0.157
**HOMA-IR**			
At week 12	3.4 (0.9, 9.0)	2.7 (0.9, 7.4)	
Relative change (%)	10.9 (-68.8, 201.3)	-3.1 (-64.8, 108.2)	0.928
**HOMA-β**			
At week 12	45.4 (13.7, 230.4)	20.2 (9.2, 72.0)	
Relative change (%)	68.9 (-59.5, 399.2)	-1.4 (-63.2, 116.7)	0.001**
**Total cholesterol (mg/dL)**			
At week 12	152.0 (90.0, 213.0)	172.0 (105.0, 238.0)	
Relative change (%)	-21.6 (-38.9, 8.1)	-5.6 (-27.8, 58.2)	0.004**
**HDL-C (mg/dL)**			
At week 12	41.0 (27.0, 59.0)	43.0 (30.0, 56.0)	
Relative change (%)	-9.3 (-26.2, 13.9)	-3.1 (-18.6, 20.5)	0.059
**LDL-C (mg/dL)**			
At week 12	86.0 (44.0, 152.0)	107.5 (44.0, 170.0)	
Relative change (%)	-17.4 (-54.1, 48.3)	-5.7 (-27.9, 83.6)	0.023*
**Triglyceride (mg/dL)**			
At week 12	109.0 (44.0, 347.0)	180.0 (49.0, 286.0)	
Relative change (%)	-29.5 (-60.7, 32.0)	5.2 (-67.8, 59.5)	0.004**
**Triglyceride/HDL-C**			
At week 12	2.4 (1.3, 10.2)	4.5 (1.0, 9.5)	
Relative change (%)	-25.8 (-62.3, 27.5)	0.2 (-64.9, 85.9)	0.051
**Score of TCM symptoms of diabetes**			
At week 12	1.0 (0.0, 9.0)	3.0 (0.0, 9.0)	
Relative change (%)	-80.0 (-0.0, 16.7)	-58.6 (-100.0, 66.7)	0.107

The data are presented as the median (min, max). Statistics were evaluated by using the Mann–Whitney U test for continuous variables (*p < 0.05, **p < 0.01). After 12 weeks of YH1 treatment, there were significant reductions in body weight, waist circumference, HbA_1c_, 2hPG, triglyceride, total cholesterol, and LDL-C. A significant increase in HOMA-β was observed in the YH1 group. There were trends of decreased FBG and TG/HDL-C levels in the YH1 group, but the differences were not significant. Relative changes in HOMA-IR and TCM symptom scores showed no significant differences between groups. HbA_1c_, glycated hemoglobin; FPG, fasting plasma glucose; 2hPG, 2-hour postprandial glucose; HOMA-IR, homeostatic model assessment of insulin resistance; HOMA-β, homeostatic model assessment of β cell function; HDL-C, high-density lipoprotein cholesterol; LDL-C, low-density lipoprotein cholesterol; TCM, traditional Chinese medicine.

HOMA for IR and β-cell function was evaluated. The respective changes in the median HOMA-β score from baseline were 68.9% and -1.4% for the YH1 and placebo groups, representing a significant between-group difference (*p* = 0.001). However, neither a significant reduction in FPG level (*p* = 0.066) nor a significant change in HOMA-IR score was observed between the two groups. Regarding lipid metabolism, the YH1 group showed a 21.6% reduction in total cholesterol, a 17.4% reduction in LDL-C, and a 29.5% reduction in triglycerides, all of which were significant compared with the corresponding values in the placebo group (Table 3). However, HDL-C levels were not considerably altered in either group. The anthropometric characteristics of body weight and waist circumference decreased significantly by 0.5% and 1.1%, respectively, in the YH1 group compared with the placebo group (Table 3). No significant differences in symptoms of polyphagia and hunger were observed between the groups (S1B Table).

### Safety results

No serious adverse events were reported in the YH1 and placebo groups (Tables 4 and 5). A total of 41 participants completed this 12-week trial. Blood pressure and heart rates were recorded at week 12 without abnormal changes in either group. Liver and kidney function was monitored before and after this trial. No significant changes in plasma ALT or creatinine were observed in either group (Table 4).

**Table 4:** Safety indicators in the YH1 and placebo groups.

Safety Indicators at Week 12	YH1 Group (*n* = 21)	Placebo Group (*n* = 20)	*p* value
	median (min, max)	median (min, max)	
Systolic BP (mmHg)	132.0 (111.0, 150.0)	126.0 (97.0, 167.0)	.22
Diastolic BP (mmHg)	75.0 (64.0, 89.0)	73.5 (52.0, 100.0)	.31
Heart rate (beats/min)	82.0 (69.0, 108.0)	80.5 (61.0, 111.0)	.61
ALT (U/L)	27.0 (7.0, 76.0)	24.5 (14.0, 49.0)	.87
Creatinine (mg/dL)	0.69 (0.34, 1.00)	0.62 (0.36, 1.13)	.81

To assess the relative change in numerical data between groups, the Mann–Whitney U test was applied to determine significance. No significant changes in blood pressure, heart rate, plasma ALT or creatinine were observed between the YH1 and placebo groups during the 12 weeks of treatment. BP, blood pressure; ALT, alanine aminotransferase.

**Table 5:** Adverse events in the YH1 and placebo groups.

Adverse events	YH1 Group (*n* = 23)	Placebo Group (*n* = 23)	*p* value
	n (%)	n (%)	
Diarrhea	7 (30.4)	3 (13.0)	.28
Nausea, bloating, GERD	5 (21.7)	8 (34.8)	.51
Dizziness	0	4 (17.4)	-
Hypoglycemia	2 (8.7)	0	-
Eczema	1 (4.3)	1 (4.3)	1.00
URI	4 (17.4)	3 (13.0)	1.00
Foot cellulitis	0	1 (4.3)	-
Hordeolum	0	1 (4.3)	-
Constipation	1 (4.3)	0	-

All adverse effects observed during this trial are listed. Five dropouts, including 2 subjects in the YH1 group and 3 subjects in the placebo group, were also accounted for adverse events and were reported in the safety analysis. The severities of adverse events were all below grade 2 as measured by the CTCAE grading system. Fisher’s exact test was applied for the analysis of categorical data, and there were no significant differences between groups. GERD, gastroesophageal reflux disease; URI, upper respiratory tract infection.

The analysis of adverse events included 46 subjects in this trial. The incidence of diarrhea in the YH1 and placebo groups was 30.4% and 13.0%, respectively. The severity of diarrhea was below grade 2 in all cases as measured by the CTCAE grading system. All subjects with diarrhea tolerated it well without any decrease in dosage or drug intervention. Moreover, 21.7% of patients in the YH1 group and 34.8% of patients in the placebo group had other adverse GI events, including nausea, bloating, or gastroesophageal reflux disease (Table 5), with no significant differences between the groups. The severity of the above adverse GI events was also below grade 2. YH1 or placebo treatment was withdrawn temporarily when a participant reported discomfort, including nausea and bloating. The treatment was resumed if the symptoms resolved. Two subjects in the YH1 group experienced hypoglycemia. Hypoglycemia did not occur again after dosage reduction of sulfonylureas. All participants had a medication adherence rate above 80% (S3 Table).

## Discussion

This study demonstrated that the combination of YH1 and OHAs had therapeutic value and was safe for treating patients with poorly controlled type 2 diabetes and a BMI ≥ 23 kg/m^2^. Moreover, YH1 together with OHAs also had beneficial effects on weight control and lipid metabolism. In Asia, a BMI over 23 kg/m^2^ is considered a moderate-to-high risk factor for type 2 diabetes and cardiovascular disease [18]. There are significant positive correlations between being overweight or obese and having poor glycemic control [19]. Therefore, this clinical trial selected patients whose diabetes was poorly managed with at least three classes of OHAs, who were overweight or obese, and whose HbA_1c_ persisted at > 7.0% (53 mmol/mol) for more than six months. According to the IDF guidelines, people with type 2 diabetes are advised to maintain HbA_1c_ levels below 7.0% (53 mmol/mol) to minimize the risk of developing complications. Numerous patients with type 2 diabetes still have poor glycemic control after receiving several classes of OHAs or newer and more expensive agents including dipeptidyl peptidase-4 (DPP-4) inhibitors and sodium-glucose cotransporter 2 inhibitors. Insulin treatment is suggested when optimized OHAs and lifestyle interventions are unable to maintain target glucose control [2]. Although biosimilar insulin therapy is not uncommon in Asia, only one-third of patients with type 2 diabetes in Asia and 7.4% of patients with type 2 diabetes in Taiwan achieve HbA_1c_ < 7.0% (53 mmol/mol) after 6 months of basal insulin therapy [20, 21]. An alternative option is to add a glucagon-like peptide-1 receptor agonist (GLP-1 RA). However, GLP-1 RAs are associated with adverse GI effects, especially nausea and vomiting. The route of administration, availability, and cost of GLP-1 RAs raise concerns. This study proposes a new add-on oral medication YH1 to achieve better glycemic control in treatment-resistant type 2 diabetes.

In a previous clinical trial [22], 500 mg of berberine 3 times daily was prescribed to treat 48 adults who had poorly controlled type 2 diabetes under a single or combination treatment regimen including metformin, sulfonylureas, acarbose, and insulin therapy. After cotreatment with berberine and the existing therapy for 3 months, the percentage change in HbA_1c_ from baseline was a 9.9% reduction. Significant decreases in FBG and 2hPG levels were also reported. The hypoglycemic effect of berberine in that study was probably an effect of enhanced insulin sensitivity. The HOMA-IR level was decreased by nearly 50% without a significant change in HOMA-β. In this study, patients with poorly controlled type 2 diabetes who received at least 3 classes of OHAs were prescribed 6 g of YH1 3 times daily; this daily dose of YH1 contains 360.9 mg of berberine. Surprisingly, although the present YH1 treatment had a 4-fold lower berberine dosage than that used in the previous study [22], the present YH1 treatment achieved a more noticeable reduction in HbA_1c_ of 11.1%. This finding implied that the hypoglycemic effect of YH1 is better than that of a single pure compound. The 2hPG was significantly reduced in the YH1 group. YH1 and GLP-1 RAs may share a similar mechanism in which the hypoglycemic effect depends on the blood glucose concentration. Therefore, YH1 did not cause hypoglycemia when used alone.

Regarding anthropometric characteristics, the body weight and waist circumference declined significantly after 3 months of YH1 and OHA treatment. Previous studies also reported that the waist circumference and waist-hip ratio of patients were significantly decreased after berberine treatment [22]. Both reductions may be related to the effect of berberine on fat distribution or GLP-1 RA. In addition, a systematic review reported that berberine significantly decreased serum triglycerides, total cholesterol, and LDL-C [23]. The lipid-lowering effect of berberine was mainly due to a decrease in intraluminal cholesterol micellarization, enterocyte cholesterol uptake and secretion, AMPK activation, LDL receptor upregulation, mitogen-activated protein kinase/extracellular signal regulated kinase (MAPK/ERK) pathway blockage and proprotein convertase subtilisin/kexin type 9 inhibition; these effects differ from the mechanism of statin drugs [13, 24, 25]. Our study showed that YH1 had similar lipid-lowering effects on lipid metabolism by reducing serum triglycerides, total cholesterol, and LDL-C. Whether YH1 achieved its lipid-lowering effect through the abovementioned mechanisms requires further investigation. There was a decreasing trend in HDL-C in the YH1 group but no significant difference between groups. A similar result was reported in the berberine study [22], but further research is still required. In addition, a trend of reduction in TG/HDL-C after 12 weeks of YH1 treatment was found, as shown in Table 3. This result implies that YH1 may reduce the risk of coronary atherosclerosis progression in patients with poorly controlled type 2 diabetes and a BMI ≥ 23 kg/m^2^. Interestingly, YH1 treatment had a lesser effect on insulin sensitivity and the decrease in FBG level, but these effects were not significant in this trial. This finding may be due to the small sample size or a dose-dependent phenomenon of berberine.

With respect to safety, neither serious adverse events nor abnormal hepatic and renal function were recorded during this clinical trial. First, hypoglycemia events occurred in two subjects in the YH1 group. HbA_1c_ levels decreased from 8.1% (65 mmol/mol) to 7.2% (55 mmol/mol) and from 7.7% (61 mmol/mol) to 6.8% (51 mmol/mol) in these two subjects. There is no direct evidence that a component of YH1 causes hypoglycemia. After reducing the dosage of sulfonylureas in these two patients, no further hypoglycemic events were observed. Second, research has revealed that patients with type 2 diabetes have an altered intestinal microbiota characterized by a decrease in the *Bacteroidetes*/*Firmicutes* ratio [26]. Berberine had an effect on the enrichment of short-chain fatty acid (SCFA)-producing bacteria, especially butyrate-producing bacteria. The modulation of the gut microbiota may contribute to beneficial effects in the host [27]. SCFA receptors are expressed in pancreatic beta cells, enteroendocrine cells, immune cells, white adipocytes, and other cells. Through SCFA receptors, gut microbiota-derived SCFAs have effects on insulin secretion, GLP-1 release, energy expenditure, and the regulation of inflammatory responses [28].

GI adverse events resulting from berberine, including diarrhea, constipation, flatulence, and abdominal pain have been reported in previous clinical studies [22, 29]. Similarly, GI adverse events including diarrhea, nausea, bloating, gastroesophageal reflux disease, and constipation were observed during 12-week YH1 and OHA treatments. However, the incidences of diarrhea, bloating, and gastroesophageal reflux disease were higher in both the YH1 and placebo groups. In light of a positive correlation between increased BMI and diarrhea [30], the diarrhea in both groups may have resulted from the patients being overweight or obese. SLBZS was reported to modulate the gut microbiota during the alleviation of antibiotic-associated diarrhea by enriching SCFA-producing bacteria [31]. Atractylenolide III, found in SLBZS, was noted to have a gastroprotective effect via inhibition of the matrix metalloproteinase (MMP)-2 and MMP-9 pathways [32]. Therefore, YH1 containing SLBZS could alleviate the negative GI effects of berberine from *Rhizoma Coptidis*. Participants tolerated the adverse events well, without any decrease in YH1 dosage. A further investigation involving microbiome assessments of fecal samples from YH1-treated objects may directly identify the intestinal microbiota population and alteration.

The current study has the following limitations. First, the sample size was small due to difficulties in enrolling patients. Larger, multicenter randomized controlled trials are warranted to ensure the efficacy and safety of YH1. Second, this study enrolled overweight or obese participants, and, consequently, the results may not be applicable to normal or underweight patients with type 2 diabetes. Finally, the 12-week study period was relatively short. Many participants did not achieve the HbA_1c_ target of < 7.0% (53 mmol/mol), likely due to the insufficient duration of treatment. Longer treatment and follow-up periods are required to evaluate the long-term effects and safety of YH1.

In conclusion, this randomized double-blind placebo-controlled pilot study is the first to demonstrate that concentrated Chinese herbal extract granules (namely, YH1) in combination with OHAs can benefit patients with poorly controlled type 2 diabetes and a BMI ≥ 23 kg/m^2^. In contrast to insulin treatment, the combination of YH1 and OHAs avoided the side effects of hypoglycemia and weight gain. After 12 weeks of YH1 and OHA treatment, there were significant reductions in body weight, waist circumference, 2hPG, HbA_1c_, triglycerides, total cholesterol, and LDL-C. There was also a significant increase in HOMA-β. Therefore, YH1 is a safe add-on medication for OHAs and provides therapeutic effects for overweight or obese patients with poorly controlled type 2 diabetes. In the future, a larger study population with longer treatment and follow-up periods will be required for further verification.

## Supporting information

S1 CONSORT checklist (PDF)

S1 Protocol (PDF)

S1 Fig Protocol for chemical analysis and the 2D-HPLC fingerprint of YH1. (PDF)

S1 Table Scores of TCM symptoms of diabetes and changes in scores. (PDF)

S2 Table Baseline classification of antidiabetic agents. (PDF)

S3 Table Medication adherence rate. (PDF)
