# Supplementary material for: The efficacy and safety of concentrated herbal extract granules, YH1, as an add-on medication in poorly controlled type 2 diabetes: A randomized, double-blind, placebo-controlled pilot trial
Source: PLoS One. 2019 Aug 15;14(8):e0221199. doi: 10.1371/journal.pone.0221199 (PMC6695147; doi:10.1371/journal.pone.0221199)
Supplement: S1 Protocol — (PDF) [file pone.0221199.s002.pdf]

長 庚 醫 學 研 究 計 畫 申 請 書

(Application of Chang Gung Memorial Hospital Research Program)

基本資料(Personal information)

<table>
<tr><td rowspan="2">計 畫 類 別
(Project category)</td><td rowspan="2">■個別型計畫
(Individual Plans)
□整合型計畫</td><td>整合型總計劃主持人</td><td></td></tr>
<tr><td>整合型總計劃名稱</td><td></td></tr>
<tr><td>計 畫 類 別
(Project category)</td><td colspan="3">■醫學類(Medicine) □管理學類 □教學類 □實驗室維持 □廠商部份贊助
□社區服務 □其他</td></tr>
<tr><td>計 劃 性 質
(Project characteristic)</td><td colspan="3">□基礎醫學研究 ■臨床醫學研究(Clinical Medical Research)
□臨床訓練性(動物實驗) □生物科技研究</td></tr>
<tr><td rowspan="2">主 持 人
Principal investigator (PI)</td><td colspan="3">姓名(Name)：黃悅翔 (Yueh-Hsiang Huang)
服務單位(Service)：中醫部中醫內兒科
(Department of Traditional Chinese Medicine)</td></tr>
<tr><td colspan="3">職稱：講師 貢獻比：30%</td></tr>
<tr><td rowspan="2">共同主持人
(co-PI)</td><td colspan="3">姓名(Name)：吳宜鴻 (Yi-Hong Wu)
服務單位(Service)：中醫部中醫內兒科
(Department of Traditional Chinese Medicine)</td></tr>
<tr><td colspan="3">職稱：助理教授 貢獻比：13%</td></tr>
<tr><td rowspan="2">共同主持人
(co-PI)</td><td colspan="3">姓名(Name)：陳思達 (Szu-Tah Chen)
服務單位(Service)：內科部新陳代謝科
(Division of Endocrinology and Metabolism, Department of Internal Medicine)</td></tr>
<tr><td colspan="3">職稱：助理教授 貢獻比：13%</td></tr>
<tr><td rowspan="2">共同主持人
(co-PI)</td><td colspan="3">姓名(Name)：劉鳳炫 (Feng-Hsuan Liu)
服務單位(Service)：內科部新陳代謝科
(Division of Endocrinology and Metabolism, Department of Internal Medicine)</td></tr>
<tr><td colspan="3">職稱：助理教授 貢獻比：13%</td></tr>
</table>

<table>
<tr><td colspan="2">共同主持人
(co-PI)</td><td>姓名(Name)：林志榮 (Jr-Rung Lin)
服務單位(Service)：長庚大學臨床資訊與醫學統計研究中心
(Clinical Informatics and Medical Statistics Research Center and Graduate Institute of Clinical Medicine, Chang Gung University)</td></tr>
<tr><td colspan="2"></td><td>職稱：助理教授 貢獻比：13%</td></tr>
<tr><td colspan="2">共同主持人
(co-PI)</td><td>姓名(Name)：謝勝湖 (Sheng-Hwu Hsieh)
服務單位(Service)：內科部新陳代謝科
(Division of Endocrinology and Metabolism, Department of Internal Medicine)</td></tr>
<tr><td colspan="2"></td><td>職稱：助理教授 貢獻比：13%</td></tr>
<tr><td colspan="2">共同主持人
(co-PI)</td><td>姓名(Name)：劉耕豪 (Geng-Hao Liu)
服務單位(Service)：中醫部
(Department of Traditional Chinese Medicine)</td></tr>
<tr><td colspan="2"></td><td>職稱：講師 貢獻比：5%</td></tr>
<tr><td rowspan="2">計劃
名稱
(Project Name)</td><td>中文</td><td>中藥 YH1 作為附加藥物對於血糖控制不佳的第 2 型糖尿病患之療效與安全評估：隨機、雙盲、安慰劑對照試驗</td></tr>
<tr><td>英文</td><td>The Efficacy and Safety of Chinese Herbal Medicine YH1 as Add-On Medication in Poorly Controlled Type 2 Diabetes Patients: A Randomized Double-Blind Placebo-Controlled Trial</td></tr>
<tr><td colspan="2">執行期限
(Execution period)</td><td>全程計劃：自民國 105 年 03 月 01 日起至民國 107 年 02 月 28 日
本年度計劃：自民國 105 年 03 月 01 日起至民國 107 年 02 月 28 日
(Between March 2016 and February 2018)</td></tr>
<tr><td colspan="2">研究學門/審查組別
(Research Gate / Review Group)</td><td>研究學門(Research Gate)：中醫藥 (Chinese herbal medicinie)
審查組別(Review Group)：藥學及中醫藥 (Pharmacy and Chinese Medicine)</td></tr>
<tr><td colspan="3">本年度申請主持長庚研究計畫共 1 件。(共同主持之計畫不予計入)
本件在本年度所申請之計畫中優先順序(不得重複)為第 1 。</td></tr>
<tr><td colspan="3">本計畫是否為跨院校合作 ■否； □是，合作機構：
本計畫是否為國際合作計畫 ■否； □是，合作國家：</td></tr>
</table>

主 持 人 最 近 兩 年 所 主 持 研 究 計 劃 情 形

研究計劃主題	編號	研究期限	研究報告提出/論文刊登雜誌及日期

<table>
<tr><td></td><td></td><td></td><td></td></tr>
<tr><td></td><td></td><td></td><td></td></tr>
<tr><td colspan="4">□　本計劃請勿送院外評審
□　本計劃請迴避　　　　　　　　　　　　　　　　醫師/教授 評審</td></tr>
<tr><td rowspan="2">計劃連絡人</td><td rowspan="2">黃悅翔</td><td>院/校內分機</td><td></td></tr>
<tr><td>院內院內 GSM 後五碼</td><td>60240</td></tr>
</table>

申請補助經費(Apply for subsidy)
長庚醫學研究計畫經費
(Funding of Chang Gung Memorial Hospital Research Program)：

執行年次<br>補助項目	第一年<br>(105 年 03 月～<br>106 年 02 月)	第二年<br>(__年__月～<br>__年__月)	第三年<br>(__年__月～<br>__年__月)	第四年<br>(__年__月～<br>__年__月)	第五年<br>(__年__月～<br>__年__月)
人事費					
設備費					
耗材費<br>(Consumables fee)	600,000				
貴重儀器使用費					
其他研究有關費用					
總計<br>(Total)	NT600,000				
博士後研究員人數	共<br>名	共<br>名	共<br>名	共<br>名	共<br>名

表 C002

註：為協助計畫主持人提升計畫執行及研究成果產出品質，共同主持人應對計劃執行進度及研究成果負監督及保密之責，對於研究年資小於五年之計畫主持人，共同主持人需負指導與協助之責。

計劃主持人簽章　　　　日期

共同主持人簽章　　　　日期

共同主持人簽章　　　　日期

共同主持人簽章　　　　日期

共同主持人簽章　　　　日期

計畫中文摘要(Abstract in Chinese)：

請於五百字內就本計畫要點作一概述，並依本計畫性質自訂關鍵詞。

關鍵詞：第二型糖尿病、中藥、隨機對照試驗、血糖控制

第二型糖尿病是一種嚴重影響全世界的慢性代謝性疾病，它總是位居台灣的前十大死因。至今，臨床上還有許多第二型糖尿病患者已服用三種以上口服降糖藥物仍不能有效地控制其糖化血色素。低血糖和體重增加是接受胰島素治療的常見副作用，而且在台灣許多第二型糖尿病患都不願意接受胰島素注射。目前在中國大陸的糖尿病患使用中藥治療是很普遍的，且一些治療成效也已經發表在國際期刊。在本研究中，我們將探討中藥(YH1) 作為附加用藥對於血糖控制不佳的第二型糖尿病患者是否能改善其血糖控制與評估其安全性。

預計於新陳代謝科或中醫內科門診收案 80 位血糖控制不佳的第二型糖尿病患者(HbA1c ≥ 7%)納入這項隨機、雙盲、安慰劑對照試驗。受試者將被隨機分派接受 YH1（6 克）或安慰劑，一日三次，連續 12 週。所有受試者持續接受原本的口服降血糖藥物治療且沒有任何劑量或藥物改變。在這 12 個星期內，將監控糖化血色素、空腹血糖、飯後血糖、腰圍尺寸、體重和 BMI。此外，也會評估 HOMA 胰島素阻抗（HOMA-IR）、β 細胞功能（HOMA-β）、血脂與肝腎功能。獨立的統計學者將在試驗結束後進行數據分析。

表 C005

計畫英文摘要(Abstract)：請於五百字內就本計畫要點作一概述，並依本計畫性質自訂關鍵詞。

Keywords：type 2 diabetes, Chinese herbal medicine, randomized controlled trial, glycemic control

Type 2 diabetes mellitus is a chronic metabolic disease that seriously affects patients worldwide, and it is always among the top 10 causes of death in Taiwan. To date, still many patients who take more than three kinds of oral hypoglycemic agents could not effectively control their HbA1c levels in clinics. Hypoglycemia as well as weight gain are common side effect with insulin therapy, and many patients in Taiwan are not willing to receive insulin injection. It is common for diabetic patients treated with Chinese herbal medicine in China currently, and some therapeutic effects have been published in SCI journals. In this study, we will evaluate whether Chinese herbal medicine, YH1, enhances the glycemic control and is safe as add-on medication in poorly controlled type 2 diabetes patients.

A total of 80 poorly controlled type 2 diabetes patients with HbA1c ≥ 7% from Endocrinology and Metabolism clinics or Internal Chinese Medicine clinics will be enrolled in this randomized double-blind placebo-controlled trial. Subjects will be randomly assigned to receive either YH1 (6 g) or the placebo TID for 12 consecutive weeks. All subjects in both groups will also continuously receive their OHAs without any dose or medicine change. During this 12-week period, the HbA1c, FPG, 2h PG, waist circumference, body weight, and BMI will be assessed. In addition, HOMA insulin resistance (HOMA-IR), β -cell function (HOMA- β), lipid profile, liver and renal function will also be evaluated. Independent statisticians will perform the data analysis at the end of the trial.

表 C006

研究計畫之背景及目的(Background and purpose of research project)：
請詳述本研究計畫之背景、目的、重要性以及國內外有關本計畫之研究情況，重要參考文獻等。本計畫如為整合型計畫之子計畫，請就以上各點分別述明與其他子計畫之相關性。

Background

Diabetes, especially type 2 diabetes mellitus, is a chronic metabolic disease that seriously affects patients worldwide, and it is always among the top 10 causes of death in Taiwan. It is estimated that there will be over 366 million people suffering from this disease by 2030.[1] Since the HbA1c levels are correlated with diabetic complications, as shown in the following graph, good glycemic control is critical for patients with type 2 diabetes.[2] The American Diabetes Association has suggested an HbA1C treatment goal of <7% for most patients with diabetes since 1994. However, still many patients who take oral hypoglycemic agents (OHAs) could not effectively control their HbA1c levels in clinics.

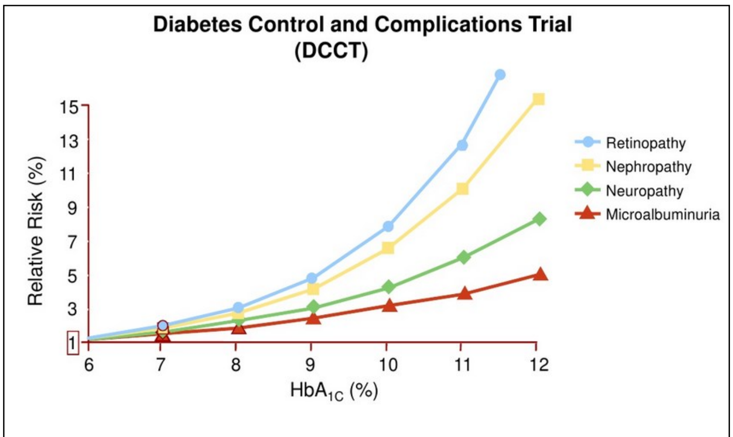

Skyler JS. Endocrinol Metab Clin North Am. 1996;25:243-254.

In addition, side effects of OHAs are increasing with dosage. For example, there are two disadvantages to metformin: the risk for lactic acidosis and its prominent gastrointestinal side effects. Common side effects associated with thiazolidinediones include edema, weight gain, macular edema and heart failure, and the main side effects of alpha-glucosidase inhibitors are flatulence and diarrhea. Dipeptidyl peptidase-4 (DPP-4) inhibitors are expensive, and recently the U.S. FDA is warning that sitagliptin, saxagliptin, linagliptin, and alogliptin may cause joint pain that can be severe and disabling.[3] Furthermore, hypoglycemia and weight gain are common side effects with sulfonylureas or insulin therapy. Due to the inconvenience and fear of injection, many type 2 diabetes patients in Taiwan are not willing to receive insulin therapy.

Traditional Chinese medicine (TCM) has a potential in the prevention and treatment of type 2 diabetes and is an excellent resource for discovering new innovative medications.[4] Although the ancients did not know diabetes as much as it

was realized in the modern world, the ancient Chinese called the diabetes-related symptoms "Xiaoke (消渴)" disease about two thousand years ago. Since then, many TCM prescriptions for Xiaoke disease have been recorded in a series of herbal classics. Even now, it is common for diabetic patients treated with TCM in China. Some therapeutic effects have been published [5-7], and studies about berberine and Rhizoma Coptidis in type 2 diabetes have been systematically reviewed. [8, 9]

YH1 is a formula combining Rhizoma Coptidis and Shenlingbaishu San, which is a classic formula described in the *Beneficial Formulas from the Taiping Imperial Pharmacy* about 1000 years ago. Rhizoma Coptidis was first recorded in *Shennong's Materia Medica* in the eastern Han dynasty (25–220 AD), and it has been prescribed by Chinese herbalists for various illnesses for more than 2000 years. Modern pharmacological research identified the major chemical constituents of Rhizoma Coptidis to be alkaloids, including berberine, coptisine, worenine, palmatine, jatrorrhizine, and epiberberine. Among these constituents, berberine is generally considered the primary contributor to its main bioactivities, such as antidiabetic, antibiotic, antioxidant, and anti-inflammatory properties. [9] Nevertheless, gastrointestinal adverse events of berberine had been reported. These adverse events included diarrhea in 10 percent, constipation in 7 percent, flatulence in 19 percent, and abdominal pain in 3.4 percent. [10] Shenlingbaishu San used in the clinical treatment of diabetic diarrhea is effective, and it could alleviate the gastrointestinal upset caused by Rhizoma Coptidis. Recently, some studies showed that Shenlingbaishu San could activate the p38 MAPK pathway in Kupffer cells and might be related to the release of inflammatory factors such as TNF-α, IL-1, and IL-6 in rats with nonalcoholic steatohepatitis (NASH).[11, 12] In fact, there is a near-universal association between NASH and insulin resistance.[13, 14] Thus, we will use YH1 based TCM to treat poorly controlled type 2 diabetic patients in clinical practice, and had a successful result according to the retrospective study as shown below. There was a very significant HbA1c reduction ($p<0.001$) in 39 cases after 3 months of YH1 therapy. Therefore, a randomized double-blind, placebo-controlled trial will be conducted to

confirm the anti-diabetic effect and safety of YH1 as add-on medication in poorly controlled type 2 diabetes patients.

Subject characteristics	YH1 (n=39)
Age (yr-old)	59.5±8.9
Male (%)	22 (56.4)
Weight (kg)	68.4±13.6
BMI	26.3±4.3
Category of OHA (n=20)	0(4), 1(5), 2(5), 3(5), 4(1)
Insulin therapy (n=37)	No(34), Yes(3)
Baseline HbA1c (%)	9.1±1.6
3m HbA1c (%)	7.8±1.4
Changes in HbA1c (%)	-1.3±1.0

References:

1. World Health Organization. Country and regional data on diabetes. Available: http://www.who.int/diabetes/facts/world_figures/en/. Accessed 28 November 2014
2. Skyler, J.S., Intensive Glycemic Control and the Prevention of Cardiovascular Events: Implications of the ACCORD, ADVANCE, and VA Diabetes Trials: A Position Statement of the American Diabetes Association and a Scientific Statement of the American College of Cardiology Foundation and the American Heart Association: Response to Lund and Vaag. Diabetes Care, 2009. 32(7): p. e92-e93.
3. DPP-4 Inhibitors for Type 2 Diabetes: Drug Safety Communication - May Cause Severe Joint Pain. http://www.fda.gov/Safety/MedWatch/SafetyInformation/SafetyAlertsforHumanMedicalProducts/ucm460238.htm.
4. Tong, X.-L., et al., Treatment of Diabetes Using Traditional Chinese Medicine: Past, Present and Future. The American Journal of Chinese Medicine, 2012. 40(05): p. 877-886.
5. Lian, F.M., et al., The Efficacy and Safety of Chinese Herbal Medicine Jinlida as Add-On Medication in Type 2 Diabetes Patients Ineffectively Managed by Metformin Monotherapy: A Double-Blind, Randomized, Placebo-Controlled, Multicenter Trial. Plos One, 2015. 10(6).
6. Lian, F.M., et al., Chinese Herbal Medicine Tianqi Reduces Progression From Impaired Glucose Tolerance to Diabetes: A Double-Blind, Randomized, Placebo-Controlled, Multicenter Trial. Journal of Clinical Endocrinology & Metabolism, 2014. 99(2): p. 648-655.
7. Ji, L.N., et al., Efficacy and Safety of Traditional Chinese Medicine for Diabetes: A Double-Blind, Randomised, Controlled Trial. Plos One, 2013. 8(2).
8. Lan, J.R., et al., Meta-analysis of the effect and safety of berberine in the treatment of type 2 diabetes mellitus, hyperlipemia and hypertension. Journal of Ethnopharmacology, 2015. 161: p. 69-81.
9. Wang, H., et al., The Antihyperglycemic Effects of Rhizoma Coptidis and Mechanism of Actions: A Review of Systematic Reviews and Pharmacological Research. Biomed Research International, 2014.

10. Yin, J., H. Xing, and H. Ye, Efficacy of berberine in patients with type 2 diabetes mellitus. Metabolism-Clinical and Experimental, 2008. 57(5): p. 712-717.
11. Yang, Q.H., et al., Effects of Chaihu-Shugan-San and Shen-Ling-Bai-Zhu-San on p38 MAPK Pathway in Kupffer Cells of Nonalcoholic Steatohepatitis. Evid Based Complement Alternat Med, 2014. 2014: p. 671013.
12. Yang, Q., et al., p38 MAPK signal pathway involved in anti-inflammatory effect of Chaihu-Shugan-San and Shen-ling-bai-zhu-San on hepatocyte in non-alcoholic steatohepatitis rats. Afr J Tradit Complement Altern Med, 2014. 11(1): p. 213-21.
13. Chitturi, S., et al., NASH and insulin resistance: Insulin hypersecretion and specific association with the insulin resistance syndrome. Hepatology, 2002. 35(2): p. 373-379.
14. Yu, J., et al., Obesity, insulin resistance, NASH and hepatocellular carcinoma. Seminars in Cancer Biology, 2013. 23(6): p. 483-491.

表 C007

研究方法及進行步驟及執行進度(Research methods, steps and implementation progress)：

1.請細述本計畫採用之研究方法與原因。

2.預計可能遭遇之困難及解決途徑。

3.重要儀器之配合使用情形。

4. 一年期以上之計畫，請分年列述。

5. 如為整合型計畫，請就以上各點分別說明與其他子計畫之相關性。

Subjects and Methods

This study is a randomized, double-blind, placebo-controlled trial.

Subjects

➢ *Inclusion criteria:*

Patients from Endocrinology and Metabolism clinics or Internal Chinese Medicine clinics who meet the following criteria will be eligible for this study:

(1) 20–75 years of age;

(2) Diagnosed as type 2 diabetics based on WHO criteria [1];

(3) Body mass index (BMI) ≥ 23 kg/m2;

(4) Have been treated with ≥ 3 kinds of oral hypoglycemic agents (OHAs) with persistent (> 6 months) high HbA1c (≥ 7.0 %).

➢ *Exclusion criteria:*

Patients will be excluded from the study if they meet one of the following conditions:

(1) Type 1 diabetes, gestational diabetes, or other specific types of diabetes;

(2) Have received insulin therapy in the past three months;

(3) Have serious gastrointestinal (GI) tract diseases, such as peptic ulcers or GI tract bleeding;

(4) Experience stressful situations, including diabetic ketoacidosis, nonketotic hyperosmolar diabetic coma, severe infection, or surgery in the previous one month;

(5) Suffer from hepatic insufficiency with alanine aminotransferase (ALT) 2 times the upper limit of normal or renal insufficiency with estimated glomerular filtration rate (eGFR) < 60;

(6) Uncontrolled hypertension (blood pressure ≥ 160/100 mmHg);

(7) Mental illness, abused or addicted to alcohol, psychoactive substances or other drugs;

(8) Pregnant, lactating, or plan to become pregnant;

(9) Hemoglobin disease or chronic anemia;

(10) Have underlying conditions that could lead to poor compliance;

(11) History of cerebrovascular disease or myocardial infarction;

(12) Have undergone Chinese medicine treatment in the past two weeks.

➢ *Sample size:*

Based on our previous clinical practice using YH1 in the treatment of patients with poorly controlled type 2 diabetes, we noticed that the HbA1c level averagely reduced 1.3%. This observation was supported by a retrospective study in 39 patients, and a randomized double-blind placebo-controlled trial is required to avoid Hawthorne effect or placebo effect of YH1 therapy. Therefore, we need to enroll 40 subjects per group from Endocrinology and Metabolism Clinics or Internal Chinese Medicine Clinics after screening to complete this study.

Study medication

The YH1 in one batch number will be used, manufactured by Sun Ten Pharmaceutical Co., LTD., which is a renowned GMP manufacturer of concentrated herbal extracts conforming to international standards. This herbal drug will be prepared in small granules. The YH1 consists of 12 concentrated herbal extracts, including white beans, ginseng, poria, atractylodes macrocephala, licorice, dioscorea opposite, lotus seeds, platycodon grandiflorus, coix lacryma-jobi, fructus amomi, ziziphus jujube, and coptidis rhizome. The granules will be packed in aluminum foil packages. The placebo is also prepared as granules by Sun Ten Pharmaceutical Co., LTD., and the packaging of the placebo will be identical to that of YH1.

Randomization and blinding

Random codes will be generated by an independent statistician. Study drugs prepared by Sun Ten Pharmaceutical Co., LTD. will be packed and numbered according to the random coding form, which will be concealed in opaque envelopes after randomization. These envelopes will not be decoded until the end of the trial. Study drugs will be provided based on the assigned numbers, which will be determined according to the visit sequence and study drug number sequence. During the trial, neither the clinicians nor the patients will be aware of the grouping. The only basis of drug distribution will be the unique drug number.

Intervention and efficacy evaluation

➢ *Intervention:*

The subjects will be randomly assigned to receive either YH1 or the placebo for 12 consecutive weeks. Subjects in both the YH1 and placebo groups will be orally administered two packages of granules (3 g / package) three times daily with warm water after meal. All subjects in both groups will also continuously receive their OHAs without any dose or medicine change. During the 12-week period, subjects will be assessed at 0, 2, 4, 8 and 12 weeks. In each session, subjects will be asked if there are any adverse events. All subjects will receive a symptom assessment (Table 1) [2], physical examination, and the compliance of the test drug administration. The HbA1c, FPG, and 2hPG will be measured at 0, 4 and 12 weeks. Body weight, BMI and waist circumference will also be monitored.

➢ *Index of efficacy:*

✧ Primary endpoint:

The primary endpoint is the change in HbA1c of the two groups after 12 weeks for this YH1 add-on trial in the type 2 diabetic subjects poorly controlled with OHAs therapy. The HbA1c level will be measured

in the Laboratory Medicine of Chang Gung Memorial Hospital by high-performance liquid chromatography.

- ✧ Secondary endpoints:

(1) Insulin resistance index (HOMA-IR) and β cell function index (HOMA-β) for two groups at 12 weeks after treatment;

(2) Fasting glucose and 2-hour post-meal blood glucose for two groups at 4 and 12 weeks after treatment;

(3) Lipid profile for two groups at 12 weeks after treatment;

(4) Clinical symptoms of patients in two groups at 12 weeks after treatment;

(5) Body weight and body mass index (BMI);

(6) Waist circumference

- ➢ *Index of safety:*

Based on previous clinical practice, the test herbal medication, YH1, was well tolerated and not associated with any safety issues. We consider the overall level of risk of the clinical study to be low. During this study, all adverse experiences will be monitored and recorded on the case report form with special notes made on the time of onset and resolution, severity, and the investigator's analysis of the relationship between the adverse experience and the test drug. Hepatic and renal function (alanine aminotransferase (ALT) and serum creatinine (Cr)) will also be followed-up at 12 weeks.

Condition and Procedures of Subjects Withdrew from Research

- ➢ *Withdraw decided by researcher:*

Subject withdrew the study indicates that the enrolled subject cannot continue research at certain condition, and researcher decides the subject to

withdraw from research process.

(1) During research, subject has severe acute and chronic complications of diabetes (diabetic ketoacidosis, hyperosmolar nonketotic syndrome, lactic acidosis, hypoglycemic coma, acute myocardial infarction, acute stroke, etc) or special physiological changes (ex. positive HCG) that he or she is inadequate to participate in research continuously.

(2) During research, subject is with poor compliance, dosage of medication is less than 70% of specified amount.

(3) During research, subject violates program rules, and takes oral administration of other hypoglycemic drugs without approval of researcher.

- *Subject withdraw research voluntarily:*

According to informed consent, subject has the right to withdraw research; if the subject does not put forward the idea of withdrawing research clearly, but losses to follow up for refusal taking medication or detection, he or she also withdraws research. The reason of withdraw should be comprehended and recorded. Such as feeling poor about curative effect; intolerable adverse effects; discontinue participating in clinical research for different reasons; economic factors; or loss to follow up without obvious reasons, etc. The history record table should be preserved for the subject who withdraws research for any reasons, the last detection result can be considered as final result, and curative efficacy and adverse effect should take full data set analysis.

Statistical analysis

Independent statisticians will perform the data analysis at the end of the trial. Data will be summarized as means ± S.D. Categorical data will be presented as frequencies. Paired-sample t-test will be performed to compare the difference within groups from baseline. Independent-sample t-test and the ANCOVA analysis with a

model that include the baseline value of the dependent variable as a covariate will be used for comparison between groups. The significance of the differences among different time points will be analyzed by repeated-measure analysis of variance. $\chi 2$ test will be used to compare the incidence of adverse events between the two groups. The level of statistical significance is set at $p < 0.05$.

Reference:
1. World Health Organization. Definition, diagnosis and classification of diabetes mellitus and its complications: report of a WHO consultation. Part I: Diagnosis and classification of diabetes mellitus. Geneva,1999.
2. Ji, L., et al., Efficacy and safety of traditional chinese medicine for diabetes: a double-blind, randomised, controlled trial. PLoS One, 2013. 8(2): p. e56703.

Table 1: Score of TCM symptoms of diabetes

Symptom	Mild（1point）	Moderate（2 point）	Severe（3 point）
Dry mouth and throat	Occasionally	Some times	Often
Fatigue	Able to do daily work	Hard to do daily work	Unable to do daily work
Polyphagia and easily hungry	Only happen before meal	Happen at any time	Happen at any time accompanied by hypoglycemia symptoms
Thirsty for drink	Increased water intake <500ml	500ml<Increased water intake <1000ml	Increased water intake >1000ml
Short of breath, lazy to talk	Happen after heavy work	Happen after daily work	Happen at any time
Vexation	Occasionally	Some times	Often
Feverish palms and soles	Occasionally	Some times	Often
Palpitation	Occasionally	Some times	Often
Insomnia	4h/day < Sleeping time < 6h/day	2h/day < Sleeping time <4h/day	Sleeping time <2h/day
Constipation	Dry stool, defecate everyday	Dry stool, defecate every 2-3days	Dry stool, defecate >every 3 days

Note: Score as “0” if there are no symptoms

表 C008
