# Supplementary material for: The efficacy and safety of concentrated herbal extract granules, YH1, as an add-on medication in poorly controlled type 2 diabetes: A randomized, double-blind, placebo-controlled pilot trial
Source: PLoS One. 2019 Aug 15;14(8):e0221199. doi: 10.1371/journal.pone.0221199 (PMC6695147; doi:10.1371/journal.pone.0221199)
Supplement: S1 Fig — (PDF) [file pone.0221199.s003.pdf]

S1 Fig. Protocol for chemical analysis and the 2D-HPLC fingerprint of YH1

(A) HPLC of 14 standards

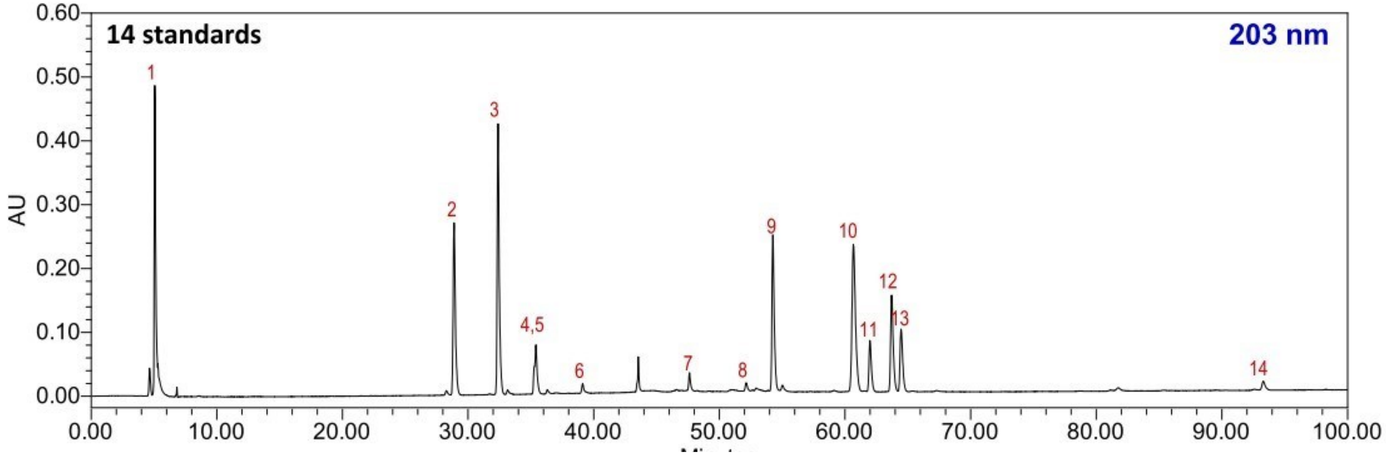

(B) HPLC of YH1

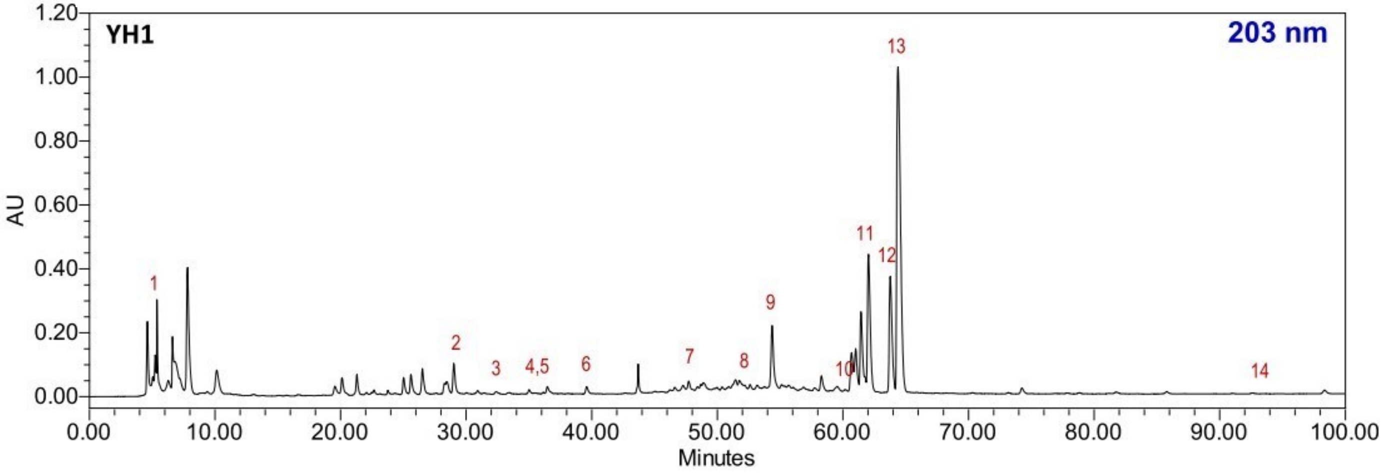

(c)

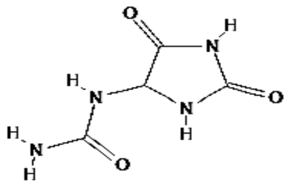

1. Allantoin

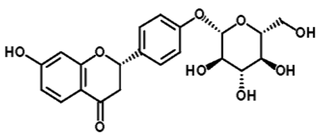

2. Liquiritin

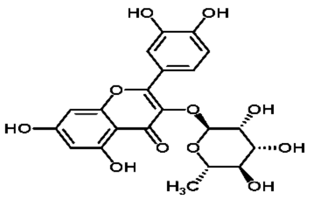

3. Quercitrin

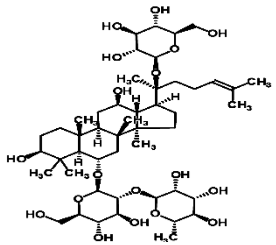

4. Ginsenoside Re

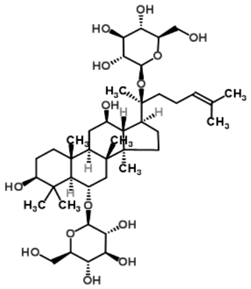

5. Ginsenoside Rg1

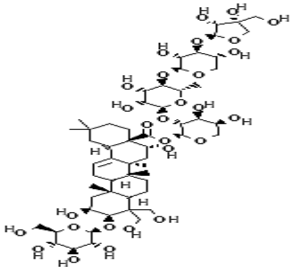

6. Platycodin D

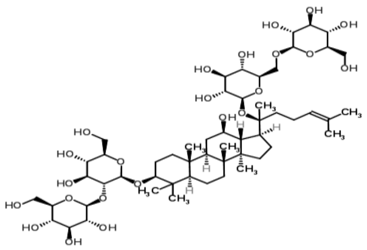

7. Ginsenoside Rb1

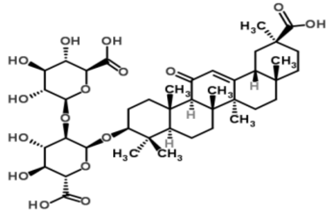

8. Glycyrrhizin

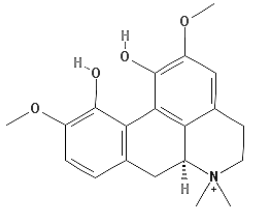

9. Magnoflorin

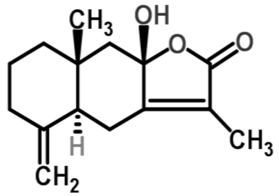

10. Atractylenolide III

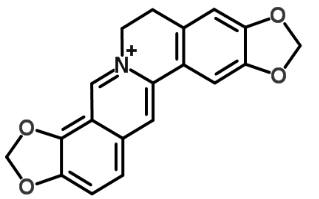

11. Coptisine

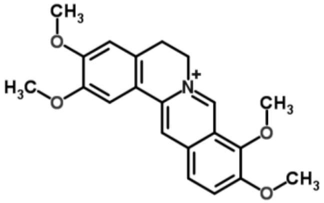

12. Palmatine

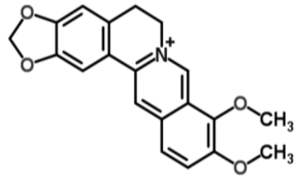

13. Berberine

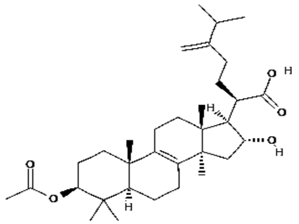

14. Pachymic acid

The chemical composition of YH1 was analysed by using a high performance liquid chromatography (HPLC) with photodiode array (PDA) detection. For preparation of sample solution, we accurately weighed the YH1 sample (0.5 g) into a suitable glass container with 20 ml of methanol/water (70/30, v/v). The sample was ultrasonicated at 25°C for 15 minutes and then shook at 160 rpm for 20 minutes in a water bath (40°C). After centrifugation and filtration, we injected 10 μl to HPLC for assay. A Biosil Aqu-ODS-W 5u column was used as the stationary phase, and a gradient of mixture of solvent A (0.1 % phosphoric acid and 0.8 g L^{-1} of sodium dodecyl sulfate in water-acetonitrile 90:10) and B (0.1 % phosphoric acid in acetonitrile) with the ratio of 100 %-0 % A and 0 %-100 % B at 0-100 minutes as the elute solution. The UV detection wavelength was set at 203 nm. The analytical run time was 100 min. Fourteen components, allantoin, atractylenolide III, berberine, coptisine, ginsenoside Rb1, ginsenoside Re, ginsenoside Rg1, glycyrrhizin, liquiritin, pachymic acid, palmatine, platycodin D, magnoflorin and quercitrin, were simultaneously qualitative analysis under the developed HPLC-PDA method. Representative HPLC of 14 standards (A) and YH1 (B) are shown. Chemical structures of identified compounds in YH1 are presented, corresponding to the peak numbers indicated in the chromatograms on the next page (C).
