# Supplementary material for: The efficacy and safety of concentrated herbal extract granules, YH1, as an add-on medication in poorly controlled type 2 diabetes: A randomized, double-blind, placebo-controlled pilot trial
Source: PLoS One. 2019 Aug 15;14(8):e0221199. doi: 10.1371/journal.pone.0221199 (PMC6695147; doi:10.1371/journal.pone.0221199)
Supplement: S1 Table — (PDF) [file pone.0221199.s004.pdf]

S1 Table. Scores of TCM symptoms of diabetes and changes in scores

(A)Score of TCM symptoms of diabetes

Symptom	Mild（1point）	Moderate（2 point）	Severe（3 point）
Dry mouth and throat	Occasionally	Some times	Often
Fatigue	Able to do daily work	Hard to do daily work	Unable to do daily work
Polyphagia and easily hungry	Only happen before meal	Happen at any time	Happen at any time accompanied by hypoglycemia symptoms
Thirsty for drink	Increased water intake <500ml	500ml<Increased water intake <1000ml	Increased water intake >1000ml
Short of breath, lazy to talk	Happen after heavy work	Happen after daily work	Happen at any time
Vexation	Occasionally	Some times	Often
Feverish palms and soles	Occasionally	Some times	Often
Palpitation	Occasionally	Some times	Often
Insomnia	4h/day < Sleeping time < 6h/day	2h/day < Sleeping time <4h/day	Sleeping time <2h/day
Constipation	Dry stool, defecate everyday	Dry stool, defecate every 2-3days	Dry stool, defecate >every 3 days

Score as “0” if there are no symptoms

(B) Change of scores in symptoms of polyphagia and easily hunger from baseline to week 12.

Change of scores	-2	-1	0	1	2
YH1 group (n)	2	13	5	1	0
Placebo group (n)	0	11	8	1	0

No significant difference was noted between groups by Fisher’s exact test (*p* value = 0.48).
