# Supplementary material for: The efficacy and safety of concentrated herbal extract granules, YH1, as an add-on medication in poorly controlled type 2 diabetes: A randomized, double-blind, placebo-controlled pilot trial
Source: PLoS One. 2019 Aug 15;14(8):e0221199. doi: 10.1371/journal.pone.0221199 (PMC6695147; doi:10.1371/journal.pone.0221199)
Supplement: S2 Table — (PDF) [file pone.0221199.s005.pdf]

**S2 Table. Baseline classification of antidiabetic agents**

| | YH1 (n=21) | Placebo (n=20) |
|---|---|---|
| Three antidiabetic drugs | 66.7 (14) | 80 (16) |
| M+SU+D | 42.9 (9) | 25 (5) |
| M+T+SG | | 5 (1) |
| M+A+SG | 4.8 (1) | |
| M+SU+SG | 14.3 (3) | 35 (7) |
| SU+T+SG | | 5 (1) |
| M+D+T | | 5 (1) |
| SU+A+SG | 4.8 (1) | 5 (1) |
| | | |
| Four antidiabetic drugs | 28.6 (6) | 20 (4) |
| M+SU+D+T | | 10 (2) |
| SU+D+T+SG | 4.8 (1) | |
| M+SU+D+A | 19.0 (4) | 5 (1) |
| M+SU+D+SG | 4.8 (1) | |
| M+SU+T+SG | | 5 (1) |
| | | |
| Five antidiabetic drugs | 4.8 (1) | |
| M+SU+D+T+A | 4.8 (1) | |

Data are presented as % (n) for categorical parameters.

M=metformin; SU= Sulfonylurea or Glinide; D= dipeptidyl peptidase 4 inhibitor; T= Thiazolidinediones; A= a-glucosidase inhibitor; SG= Sodium-glucose co-transporter-2 inhibitors
