# Supplementary material for: The efficacy and safety of concentrated herbal extract granules, YH1, as an add-on medication in poorly controlled type 2 diabetes: A randomized, double-blind, placebo-controlled pilot trial
Source: PLoS One. 2019 Aug 15;14(8):e0221199. doi: 10.1371/journal.pone.0221199 (PMC6695147; doi:10.1371/journal.pone.0221199)
Supplement: S3 Table — (PDF) [file pone.0221199.s006.pdf]

S3 Table. Medication adherence rate

Group	Patient No	Baseline to week 2 (package)	Week 3 to week 4 (package)	Week 5 to week 8 (package)	Week 9 to week 12 (package)	Total (package)	Calculated overall compliance (%)
B	1	84	84	156	166	490	97.2
A	2	84	84	168	168	504	100.0
A	3	84	84	168	168	504	100.0
B	4	84	84	168	94	430	85.3
B	5	84	84	168	168	504	100.0
A	6	76	78	134	128	416	82.5
B	7	53	84	158	160	455	90.3
A	8	74	66	124	158	422	83.7
A	10	84	84	156	158	482	95.6
B	11	82	84	166	162	494	98.0
B	12	82	82	166	168	498	98.8
B	13	84	84	162	164	494	98.0
B	14	84	66	156	168	474	94.0
A	15	84	82	164	168	498	98.8
A	16	80	84	168	168	500	99.2
B	17	72	84	168	158	482	95.6
B	18	84	82	166	162	494	98.0
A	19	68	82	138	143	431	85.5
A	20	60	84	156	137	437	86.7
B	21	70	80	130	136	416	82.5
B	22	72	58	158	154	442	87.7
A	23	84	80	168	168	500	99.2
A	24	84	84	168	164	500	99.2
A	25	84	82	156	160	482	95.6
A	26	77	84	156	162	479	95.0
B	27	82	82	156	138	458	90.9
B	28	78	82	154	168	482	95.6
B	30	76	82	168	168	494	98.0
A	31	78	82	162	160	482	95.6
A	32	76	82	158	160	476	94.4
B	34	84	84	168	168	504	100.0
A	35	76	78	142	142	438	86.9
A	36	78	78	166	162	484	96.0
A	37	76	74	167	156	473	93.8

B	38	80	84	168	168	500	99.2
B	39	84	84	168	168	504	100.0
A	40	84	84	168	168	504	100.0
A	41	84	81	168	168	501	99.4
B	43	78	72	163	162	475	94.2
B	44	80	84	166	168	498	98.8
A	46	84	72	165	153	474	94.0
